# The roles and mechanisms of SREBP1 in cancer development and drug response

**DOI:** 10.1016/j.gendis.2023.04.022

**Published:** 2023-06-22

**Authors:** Ying He, Shasha Qi, Lu Chen, Jinyu Zhu, Linda Liang, Xudong Chen, Hao Zhang, Lvjia Zhuo, Shujuan Zhao, Shuiping Liu, Tian Xie

**Affiliations:** a School of Pharmacy, Hangzhou Normal University, Hangzhou, Zhejiang 311121, China; b Key Laboratory of Elemene Class Anti-Cancer Chinese Medicines, Engineering Laboratory of Development and Application of Traditional Chinese Medicines, Collaborative Innovation Center of Traditional Chinese Medicines of Zhejiang Province, Hangzhou Normal University, Hangzhou, Zhejiang 311121, China; c Laboratory of Cancer Genomics, Division of Cellular and Molecular Research, National Cancer Centre Singapore, Singapore 169610, Singapore

**Keywords:** Cancer, Drug, Lipid metabolism reprogramming, Resistance, SREBP1

## Abstract

Cancer occurrence and development are closely related to increased lipid production and glucose consumption. Lipids are the basic component of the cell membrane and play a significant role in cancer cell processes such as cell-to-cell recognition, signal transduction, and energy supply, which are vital for cancer cell rapid proliferation, invasion, and metastasis. Sterol regulatory element-binding transcription factor 1 (SREBP1) is a key transcription factor regulating the expression of genes related to cholesterol biosynthesis, lipid homeostasis, and fatty acid synthesis. In addition, SREBP1 and its upstream or downstream target genes are implicated in various metabolic diseases, particularly cancer. However, no review of SREBP1 in cancer biology has yet been published. Herein, we summarized the roles and mechanisms of SREBP1 biological processes in cancer cells, including SREBP1 modification, lipid metabolism and reprogramming, glucose and mitochondrial metabolism, immunity, and tumor microenvironment, epithelial–mesenchymal transition, cell cycle, apoptosis, and ferroptosis. Additionally, we discussed the potential role of SREBP1 in cancer prognosis, drug response such as drug sensitivity to chemotherapy and radiotherapy, and the potential drugs targeting SREBP1 and its corresponding pathway, elucidating the potential clinical application based on SREBP1 and its corresponding signal pathway.

## Introduction

Sterol regulatory element-binding transcription factor 1 (SREBP1) is a critical transcription factor in adipogenesis, regulating genes related to cholesterol biosynthesis, fatty acid (FA) synthesis, and lipid homeostasis. SREBP1 binding element (sterol reaction element) is present in the promoter region of genes, involved in the biosynthesis of cholesterol, FAs, and lipids, and are over-expressed in various cancers, including liver, breast, prostate, and bladder cancer. Furthermore, three subtypes of SREBPs are found in mammals. SREBP1a and SREBP1c are generated by the SREBF1 gene, with differences at their extreme N-terminals, while SREBP2 is encoded by the SREBF2 gene.^1^ Recent evidence suggests the potential role of SREBP1 in cancer occurrence and development, particularly in rapidly proliferating cancer cells. SREBP1c is the predominant SREBP1 in most adult tissues, except in some rapidly proliferating tissues.^2^

Post-translational modifications (neddylation, phosphorylation, and symmetrical dimethylarginine modification) affect the stability and bio-function of SREBP1 in various cancers. Among them, the ubiquitin-like protein nedd8 (NEDD8) binds to the substrate following a successive cascade of activating enzyme E1, conjugating enzyme E2 and ligase E3, triggering post-translational modification of this protein by neddylation, and thereby altering the activity of the target protein, subcellular localization, and protein stability.^3^ It is reported that UBC12, as an enzyme E2, is involved in adding NEDD8 to SREBP1, resulting in up-regulation of SREBP1 stability and decline in ubiquitination. Further analysis found that the three proteins (UBC12, NEDD8, and SREBP1) were up-regulated in hepatocellular carcinoma (HCC) compared to non-tumorous tissue. The SREBP1expression level positively correlates with UBC12.^4^ Moreover, SREBP1 is also modulated by phosphorylation, regulating a broad spectrum of biological processes,^5^ and symmetric dimethylarginine (SDMA) modification is regulated by protein arginine methyltransferases (PRMTs).^6^

Lipid and glucose metabolic reprogramming (Warburg effect) is important in cancer occurrence and development as they activate oncogenes and silence tumor suppressor genes.^7^^,^^8^ The main source for *de novo* lipid synthesis is glucose, and SREBP1 regulates glucose metabolism and lipid metabolic pathways. SREBP1 is a significant regulatory factor of cholesterol biosynthesis, FA synthesis, and lipid homeostasis. The activation of SREBP1 closely correlates to the glucose supply in pathophysiological conditions.^9^

Here, we reviewed and summarized the roles and molecular mechanisms of SREBP1 in tumor occurrence and development, including lipid metabolism and reprogramming, glucose and mitochondrial metabolism, epithelial–mesenchymal transition (EMT), immune and tumor microenvironment (TME), apoptosis, cell cycle, and ferroptosis. This study also described the roles of SREBP1 in cancer prognosis and drug response, such as the sensitivity to chemotherapy and radiotherapy drugs. Furthermore, we also introduced some potential drugs targeting SREBP1, shedding light on the potential application in cancer therapy based on SREBP1 and the corresponding signal pathway.

## SREBP1 modification

### Neddylation

**Figure 1:**
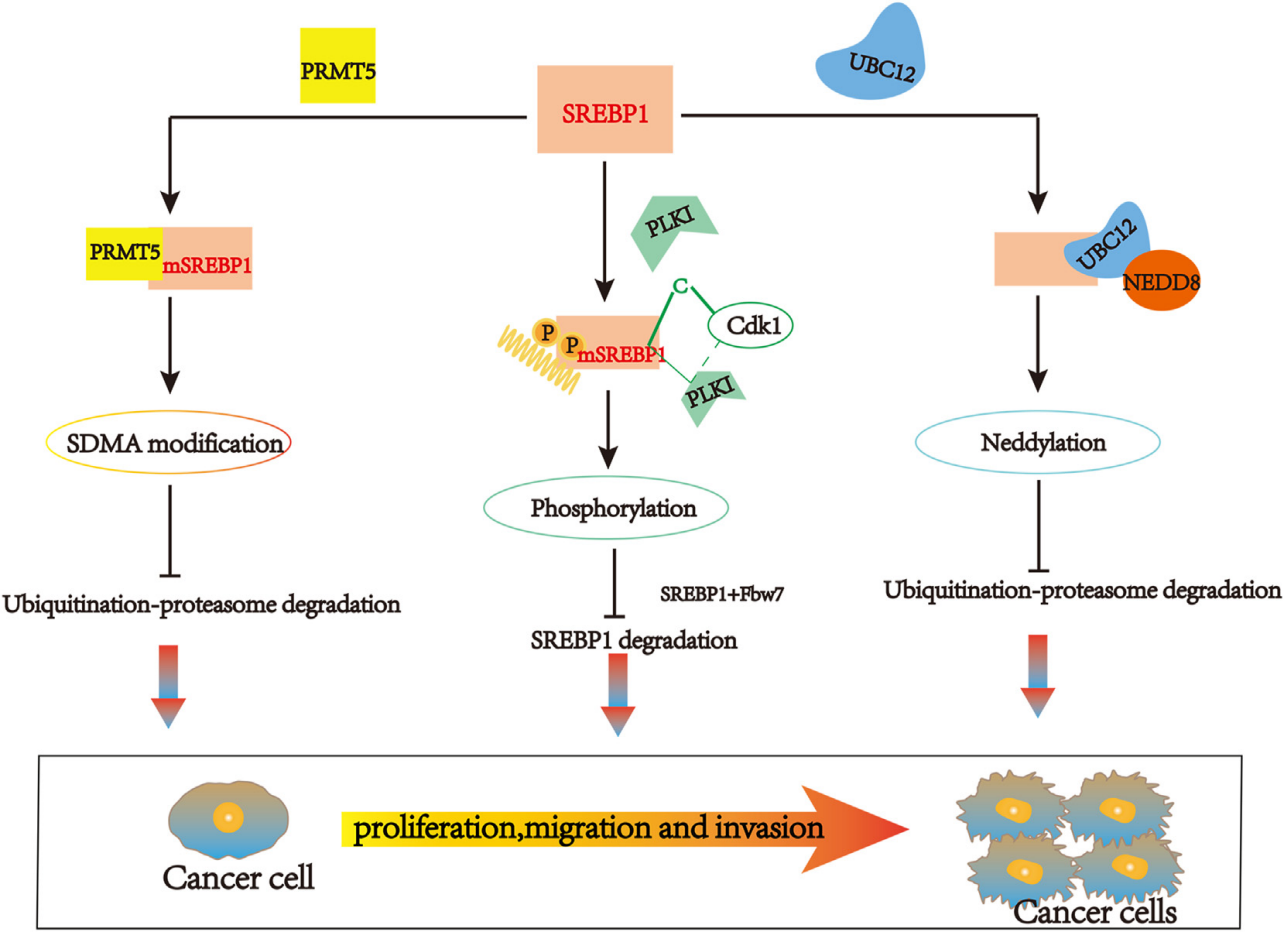
A schematic illustration of SREBP1 modifications. All three types of protein modifications can prevent the degradation of SREBP1 and enhance the stability of SREBP1, resulting in increasing malignant activity in cancer cells.

Neddylation, similar to ubiquitination and SUMOylation, is regulated by NEDD8-activating enzyme E1, NEDD8-conjugating enzyme E2, and NEDD8 ligase-like protein E3.^10^ SREBP1 is stabilized after neddylation by E2 enzyme UBC12 (Fig. 1), as a cancer gene in breast cancer and HCC. Both are up-regulated in these two types of tumor tissues, especially higher in the metastatic ones, and negatively correlated with the overall patient survival rates, separately. Meanwhile, MLN4924 (an inhibitor of E1) treatment and UBC12 knockdown could prevent SREBP1 neddylation and tumor cell phenotype change.^4^ In addition, NEDD8 overexpression promotes proliferation, invasion, and migration in cancer cells.^4^

### Protein phosphorylation and ubiquitination

Protein phosphorylation is the most common type of post-translational modification, impacting every basic cellular process.^11^ Recent evidence reveals the role and mechanism of lipid metabolism in the growth of cancer cells. SREBP1 is reportedly activated in the cellular transformation of Ras/PI3K mutants, while the reduction of SREBP1 in cancer cells attenuates cell proliferation.^12^ SREBP molecules with transcriptional activity are rapidly degraded in a phosphorylation-dependent manner by Fbw7, degrading several cell cycle proteins.^13^ Meanwhile, the protein kinase Plk1 participates in many mitotic processes, such as mitotic entry, bipolar spindle assembly, and mitotic exit.^14^^,^^15^ Phosphorylation of SREBP1c-terminal S439/415 initiated through the cyclin-dependent kinase CDK1 which is a binding site for binding to Plk1, and Plk1-mediated phosphorylation of SREBP1 decreases the degradation of SREBP1 by Fbw7, indicating that active SREBP1 stabilizes during mitosis and then affect cell division (Fig. 1). Notably, the stabilization of phosphorylation and nuclear SREBP1 during cytokinesis suggests a connection between cancer cell proliferation and lipid metabolism.^13^ However, another cyclin-dependent kinase CDK8 could enhance SREBP-1c phosphorylation, ubiquitination, and protein degradation.^16^ When nuclear SREBP1a is phosphorylated on S430 and T426 by GSK-3, Fbw7 interacts with it and promotes its ubiquitination and degradation. Besides SREBP1a, nuclear SREBP2 and SREBP1c are degraded by Fbw7.^17^

### SDMA modification

The dimethylarginines, including asymmetric dimethylarginine (ADMA) and SDMA, were first isolated from human urine in 1970.^18^ Unlike ADMA, SDMA's biological activity is reported in considerably less literature, which is another methylated form of L-arginine. PRMTs type I and II are respectively responsible for methylating only one guanidine nitrogen group (forming ADMA) or methylating both groups (forming SDMA) of L-arginine.^19^ PRMTs methylate a wide range of proteins (histones and non-histones) and affect different biological processes (differentiation, proliferation, and apoptosis) by regulating target gene transcription and protein stability. PRMT5, as a type II PRMT enzyme, mediates tumor progression by SDMA modifications of target proteins.^20^ Furthermore, PRMT5 is a novel SREBP1 regulator (Fig. 1). Liu et al reported that the mature form of SREBP1 (mSREBP1a, 68 kDa) is symmetrically dimethylated by PRMT5 on R321, thereby stabilizing SREBP1a by preventing its ubiquitination-proteasomal degradation. Compared with non-methylated mSREBP1a, methylated mSREBP1a has higher transcriptional activity, promoting tumor growth and proliferation.^21^ Meanwhile, AKT, a prominent molecule that induces malignancy and regulates tumor growth at all stages, is a promising therapeutic tumor target, and AKT overactivation is common in lung cancer.^22^ AKT activation of SREBP1 by multiple mechanisms regulates lipid metabolism in some malignancies, such as breast cancer, glioblastoma, and melanoma, making SREBP1 driven by overactive AKT a novel antitumor target to explore.^23^, ^24^, ^25^ Liu et al demonstrated that AKT was overexpressed in lung adenocarcinoma and positively correlated with SREBP1-SDMA, and AKT signaling activation could affect ubiquitin-protease degradation of SDMA-modified SREBP1, thereby increasing the stability of PRMT5-mediated mSREBP1 protein.^26^

### Lipid metabolism and reprogramming

**Figure 2:**
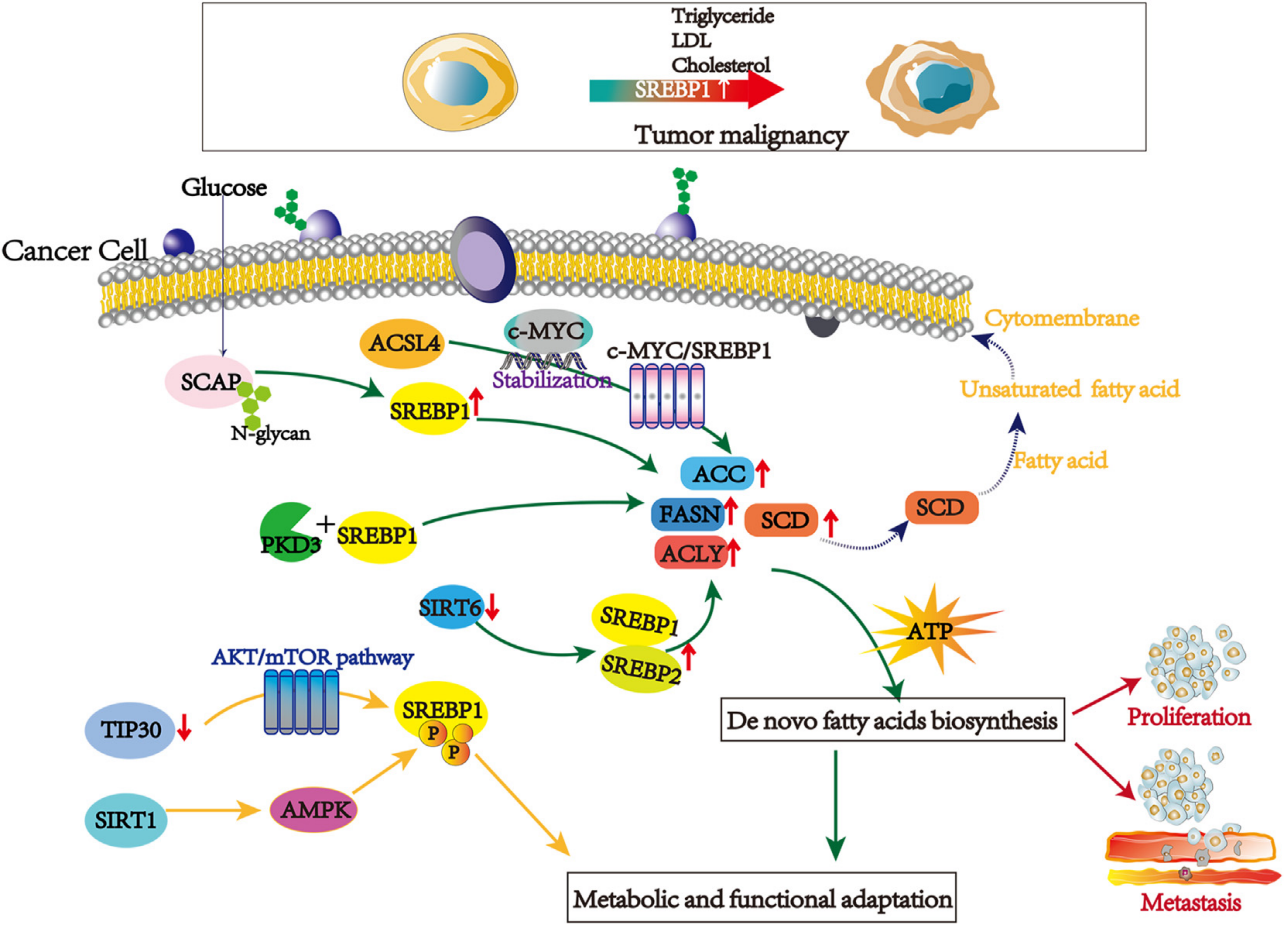
The roles and mechanisms of SREBP1 in lipid metabolism and reprogramming in cancer cells. Glucose provides the carbon source needed to synthesize lipids. Several genes and their corresponding signaling pathways promote the *de novo* synthesis of fatty acids by up-regulating SRBP1 to promote the synthesis of lipid synthetases, including ACLY, ACC, FASN, and SCD. On the other hand, lipid metabolism in cancer cells can be promoted by regulating SREBP1. The reprogramming process of lipid metabolism provides cancer cells with fatty acids, triglycerides, cholesterol, energy, and lipids for cell membrane synthesis. It indicates that the expression of SREBP1 contributes to tumor cell proliferation, migration, and invasion.

Lipids are the constituents of tissue cells and important nutrients that provide energy and essential FAs for cellular processes. As the basic components of cell membranes, they play an important part in cellular activities, such as intercellular recognition, energy supply, and signaling. Lipid metabolism is the basis of life maintenance and is divided into anabolism and catabolism. Tumor cells actuate the reprogramming of lipid metabolism mainly by influencing the uptake, synthesis, and breakdown of the three major lipid molecules such as FAs, phospholipids, and cholesterol.^27^ Lipid metabolic reprogramming usually generates signaling molecules that activate tumor-related signaling pathways and facilitate tumor cell proliferation, invasion, and metastasis, and allows tumor cells to produce more energy, facilitating tumor cell survival in a nutrient-deficient microenvironment (Fig. 2).

To support fast proliferation, *de novo* FA synthesis of lipids is up-regulated by modification of signal lipid molecules and related proteins in tumor cells, supplying materials and energy for cell membrane formation. Additionally, FA synthase (FASN) could provide novel FAs necessary for forming the plasma membrane during cell proliferation. Meanwhile, the activity and expression of FASN, ATP citrate lyase (ACLY), and acetyl-CoA carboxylase (ACC) in the biological process of synthesis from *de novo* FAs is significantly up-regulated and closely associated with the prognosis of multiple tumors (Fig. 2).^28^ Moreover, Chen et al found that ACSL4 up-regulates lipid synthases such as FASN, ACLY, SCD, and ACC through the c-Myc/SREBP1 signaling pathway, regulating the lipid metabolism reprogramming. Thus, SREBP1 is a downstream effector of ACSL4 and the basis for ACSL4-mediated HCC growth and metastasis (Fig. 2).^29^ In addition, threonine-protein kinase (PKD), as a member of the serine/threonine protein kinase family (contains PKD1, PKD2, and PKD3), is associated with many tumor biological processes such as cell proliferation, invasion, and migration.^30^^,^^31^ In prostate cancer, PKD3 significantly promotes tumor cell proliferation by modulating FASN and ACLY expression and *de novo* lipid synthesis in an SREBP1-dependent manner (Fig. 2).^32^

The lipid metabolism reprogramming allows cancer cells to produce more energy and facilitates tumor cell survival in a nutrient-deprived microenvironment. For instance, SIRT1, known as a nicotinamide adenosine dinucleotide (NAD), relies on deacetylase to remove acetyl groups from various proteins,^33^^,^^34^ and promote phosphorylation of SRBEP1 through activation of AMPK. Decreased SIRT1 can inhibit the expression and stabilization of SREBP1, inhibiting the lipid metabolism of prostate cancer cells (Fig. 2).^35^ Moreover, SIRT6, another NAD-dependent deacetylase, significantly decreases triglyceride and cholesterol production. Mechanistically, overexpression of SIRT6 results in significantly decreased expression of SREBP1 and SREBP2 and their target genes, together with the actively cleaved forms of SREBP1 and SREBP2 (Fig. 2).^36^ The tumor suppressor TIP30 (HTATIP2 or CC3) could inhibit lipid metabolism by the AKT/mTOR signaling pathway, decreasing the expression of SREBP1 and its target genes (SCD and FASN) and thus inhibiting the tumor growth of liver cells (Fig. 2).^37^

### Glucose and mitochondrial metabolism

Sugar belongs to the class of organic compounds composed of polyhydroxy aldehydes or polyhydroxy ketones and their derivatives. Glucose metabolism refers to a complex series of chemical reactions in the body involving glycogen and glucose. Glucose is the major source of lipid synthesis from scratch, and glucose consumption in cancer cells is accompanied by increased lipid synthesis.^38^ The high glucose microenvironment promotes tumor proliferation and inhibits autophagy and apoptosis by increasing the expression of SREBP1, and poor prognosis of the cancer patient is associated with elevated blood glucose levels in prostate cancer.^39^ Furthermore, glucose is critical in controlling lipid metabolism during tumorigenesis. Tumor cells induce SCAP protein N-glycosylation and promote SREBP1 activation via up-regulating glucose uptake under conditions of sterol deprivation; N-glycosylation is a key mediator of the increased SCAP level regulated by EGFR signaling and subsequent activation of SREBP1 (Fig. 2).^24^ SREBP1 protects tumor cells by enhancing glycolytic activity. SREBP1 knockout can inhibit anaerobic glycolytic activity, glucose uptake, and ATP production of HCC cells. SREBP1 also facilitates the resistance to sorafenib in HCC cells and xenograft tumors.^40^ Therefore, inhibiting glucose metabolism in tumor cell survival and development may be a crucial means of enhancing the efficacy of chemotherapeutic agents.

Mitochondrial metabolism plays a crucial role in tumor development. Mitochondrial metabolism sustains the tumor phenotype by generating and supplying essential metabolites for oncometabolite and macromolecular synthesis.^41^ Christian F Ruiz's team found that mutant KRAS promotes the *de novo* lipid synthesis and the SREBP1-mediated mitochondrial gene expression in non-small cell lung cancer, and mutant KRAS tumor cells are susceptive to FASN inhibitors. Loss of SREBP1 impairs mitochondrial metabolic function by reducing mitochondrial encoding electron transport chain genes in KRAS mutant cells. Thus, SREBP1 knockdown significantly inhibits cell growth and impairs glucose oxidative phosphorylation in mutant KRAS cells.^42^ Emerging evidence suggests that knockout of SREBP1 can reduce glycolysis, FA oxidation, and mitochondrial respiration levels, and thus inhibit xenograft tumor occurrence and growth *in vivo*.^43^

### Immunity and TME

**Figure 3:**
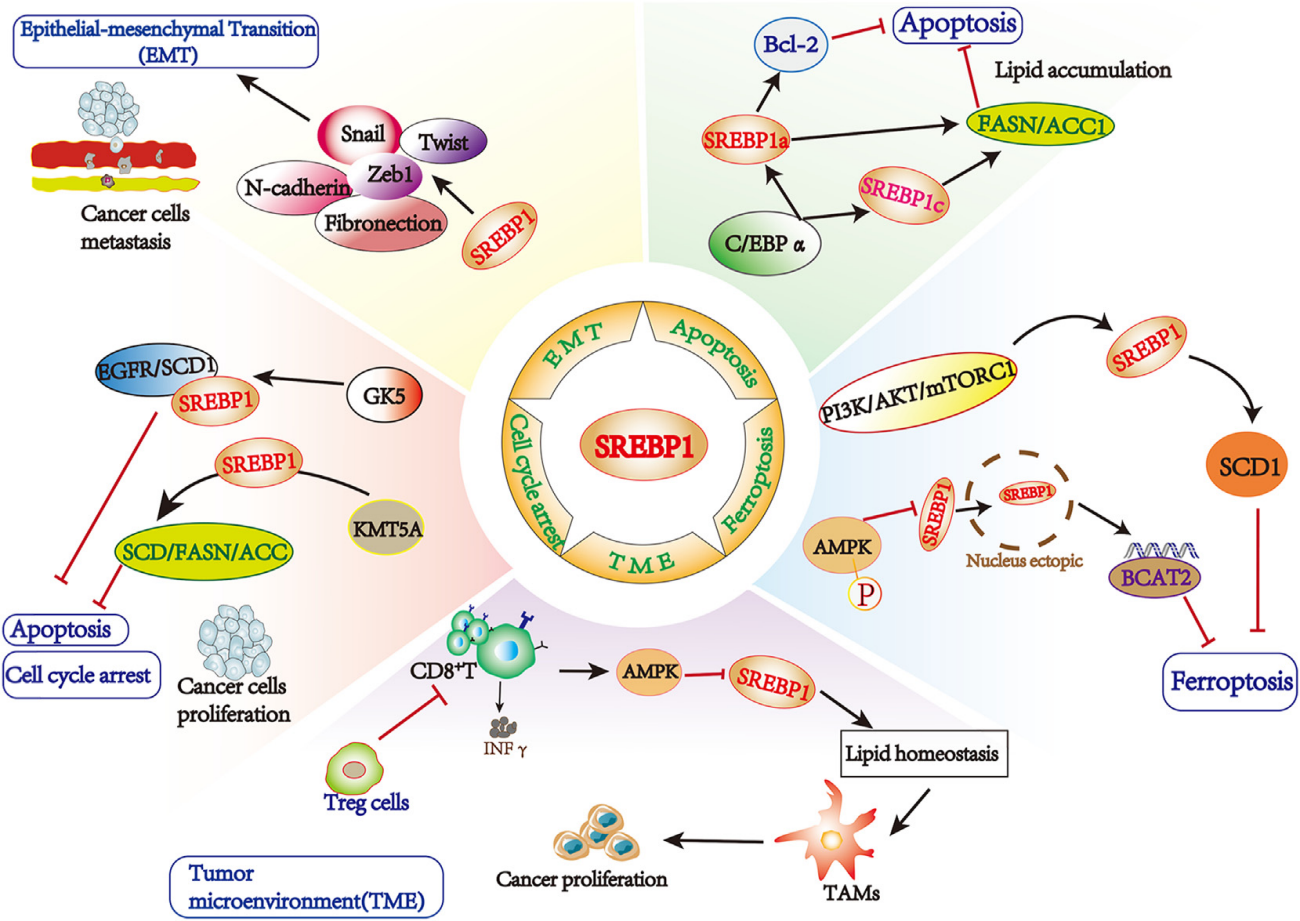
The roles of SREBP1 in several other biological processes. SREBP1 is an oncogene that inhibits the onset of apoptosis and ferroptosis of cancer cells, while promoting several biological processes such as cell cycle, EMT, and TME.

Immunotherapy targets tumor cells based on their distinct characteristics from normal cells. However, immune evasion and acquired drug resistance typically occur during treatment.^44^ Immunity response and TME play a crucial role in cancer immunotherapy. SREBP1 promotes the *de novo* FA synthesis and provides the energy for the fast proliferation of tumor cells in a nutrient-poor and oxygen-deprived environment. It has been indicated that the homeostasis of lipid storage plays a key part in promoting the survival of M2-like macrophages (TAMs) via altering SREBP1-dependent FA metabolism (Fig. 3). In the tumor-promoting microenvironment, Treg cells can indirectly promote the M2-like TAM phenotype by inhibiting interferon-γ secretion, inhibiting SREBP1-dependent FA metabolism. Therefore, targeting SREBP1 in TAMs could augment the effect of immune checkpoint blockade. Notably, AMPK can phosphorylate and inhibit SREBP activity (Fig. 3).^45^ Oishi et al demonstrated the TLR4-driven anti-inflammatory FA synthesis in an SREBP1-dependent manner, and that SREBP1 promotes transcription of TLR signaling by propelling the synthesis of anti-inflammatory FAs.^46^ In renal cancer cells, the von Hippel–Lindau tumor suppressor gene VHL could regulate SREBP1 maturation to inhibit TAG synthesis and reduce lipid accumulation; VHL can also enhance antigen processing and presentation by up-regulating MHC class and class II molecules; programmed death ligand 1 is down-regulated in VHL overexpressed cells. VHL expression correlates with SREBP1 and immunogenicity in renal tumor cells.^47^

### EMT

EMT is a process by which epithelial cells acquire mesenchymal features associated with tumorigenesis, invasion, metastasis, and resistance to treatment.^48^^,^^49^ SREBP1 knockdown could decrease EMT-relevant factors such as N-calmodulin, fibronectin, Zeb1, Snail, and Twist (Fig. 3). miR-18a-5p can bind to Snail and HDAC1/2 forming a co–repressor complex that directly targets SREBP1, regulating EMT and reduction in the metastatic ability of breast cancer cells.^50^ Though binding to the SREBP1 promoter, E2F1 could up-regulate the expression of SREBP1 and increase cell proliferation, EMT, and *de novo* FA synthesis in clear-cell renal cell carcinoma cells.^51^ Thus, targeting SREBP1-regulated EMT shows potential therapeutic implications for patients with abnormal tumor metastatic progression and lipid metabolism.

### Cell cycle

In gefitinib-induced drug-resistant cells, the knockdown of glycerol kinase (a rate-limiting enzyme) could visibly reduce the protein expression of SREBP1 and SCD1 and induce cell apoptosis and cell cycle arrest (Fig. 3).^52^ CDK family members and cyclin family members are vital for cell cycle progression. It has shown that Cdk8 and CycC could decrease the expression of lipogenic genes, lipogenesis, and lipid storage by enhancing the phosphorylation and following degradation of SREBP1 protein.^16^ In papillary thyroid cancer, inhibition of KMT5A could arrest the cell cycle in the G1/S phase by reducing the expression of SREBP1 and its downstream genes (*e.g.*, SCD, FASN, ACC) involved in lipid metabolism (Fig. 3).^53^

### Apoptosis

Apoptosis is a strictly regulated form of programmed cell death that triggers cellular self-destruction.^54^ Ionizing radiation is one of the radiation treatments in cancer therapy, which can kill tumor cells or cause serious damage to tumor cells by inducing diversified types of cell death such as apoptosis.^55^ It has been reported that mTORC1 up-regulates HIF-1α and SREBP1, followed by cardiolipin accumulation and delayed release of cytochrome C, resulting in suppressing ionizing radiation-induced apoptosis and gaining a survival advantage and radiation resistance.^56^ In valproic acid (a promising drug for cancer treatment)-treated PC-3 cells, overexpression of C/EBPα could rescue the protein levels of SREBP1, ACC1, FASN, and Bcl-2, resulting in attenuated apoptosis and lipid accumulation. Besides, both SREBP1a and SREBP1c could up-regulate FASN and ACC1 which is vital to lipid accumulation. However, only SREBP1a could observably raise the level of anti-apoptotic protein Bcl-2 (Fig. 3).^57^ Therefore, drugs such as valproic acid that target both apoptosis and lipid metabolism are a potential approach to cancer chemotherapeutics.

### Ferroptosis

Ferroptosis is an iron-dependent form of programmed cell death that is different from apoptosis, autophagy, and necrosis.^58^^,^^59^ Multi-drug resistant tumor cells, especially those in a mesenchymal and metastatic state, are highly susceptible to ferroptosis.^60^ For instance, it has been reported that continuous activation of the PI3K/Akt/mTORC1 signaling pathway generates resistance to ferroptosis in cancer by up-regulating SREBP1. Moreover, SREBP1 could inhibit ferroptosis by promoting the expression of SCD1 which is an iron-dependent enzyme involved in fatty acid desaturation.^61^ Furthermore, branched-chain amino acid transaminase 2 (BCAT2) is an aminotransferase that regulates the metabolism of sulfur amino acid and is reported to be a ferroptosis suppressor by regulating intracellular glutamate levels. Mechanistically, phosphorylation of AMPK inhibits SREBP1 nuclear translocation, and consequently inhibits the BCAT2 transcription, resulting in cancer cell ferroptosis (Fig. 3).^62^

## Roles of SREBP1 in cancer prognosis and drug response

### Potential roles of SREBP1 in cancer prognosis

SREBP1 plays a vital part in tumorigenesis and cancer progression via regulating metabolic reprogramming (lipid and glucose), immunity and TME, EMT, cell cycle, and programmed cell death. According to recent evidence, SREPB1 is the potential prognosis biomarker of cancer patients (Table 1). Taking clear cell renal cell carcinoma, for instance, higher-expression SREBP1 is correlated with poor prognosis by increasing lipid accumulation.^51^ In addition, some downstream targets of SREBP1 could also be potential diagnostic biomarkers. Highly expressed FASN regulated by SREBP1 contributes to cancer malignancy and poor prognosis in non-small cell lung cancer.^63^

**Table 1:** The expression and targets of SREBP1 in various cancer.

Types of cancer	SREBP1 regulation	Prognosis biomarker	Targets	Refs
Pancreatic cancer	Up	Negative	SREBP1/ACC/FASN/SCD1	84
Hepatocellular carcinoma	Up	Negative	UBC12/SREBP1	4
	Up	Negative	HDGF/SREBP1	85
Clear cell renal cell carcinoma	Up	Negative	E2F1/SREBP1	51
	Up	Negative	SREBP1/RNF20	86
Lung adenocarcinoma	Up	Negative	AKT/SREBP1	26
Esophageal Carcinoma	Up	Negative	SREBP1/miR-142-5p	87
	Up	Negative	PCK1/SREBP1	88
Non-Small Cell Lung Carcinoma	Up	Negative	SREBP-1/SCAP	89
Breast cancer	Up	Negative	SREBP1	90

### Chemotherapy and radiotherapy drugs

**Figure 4:**
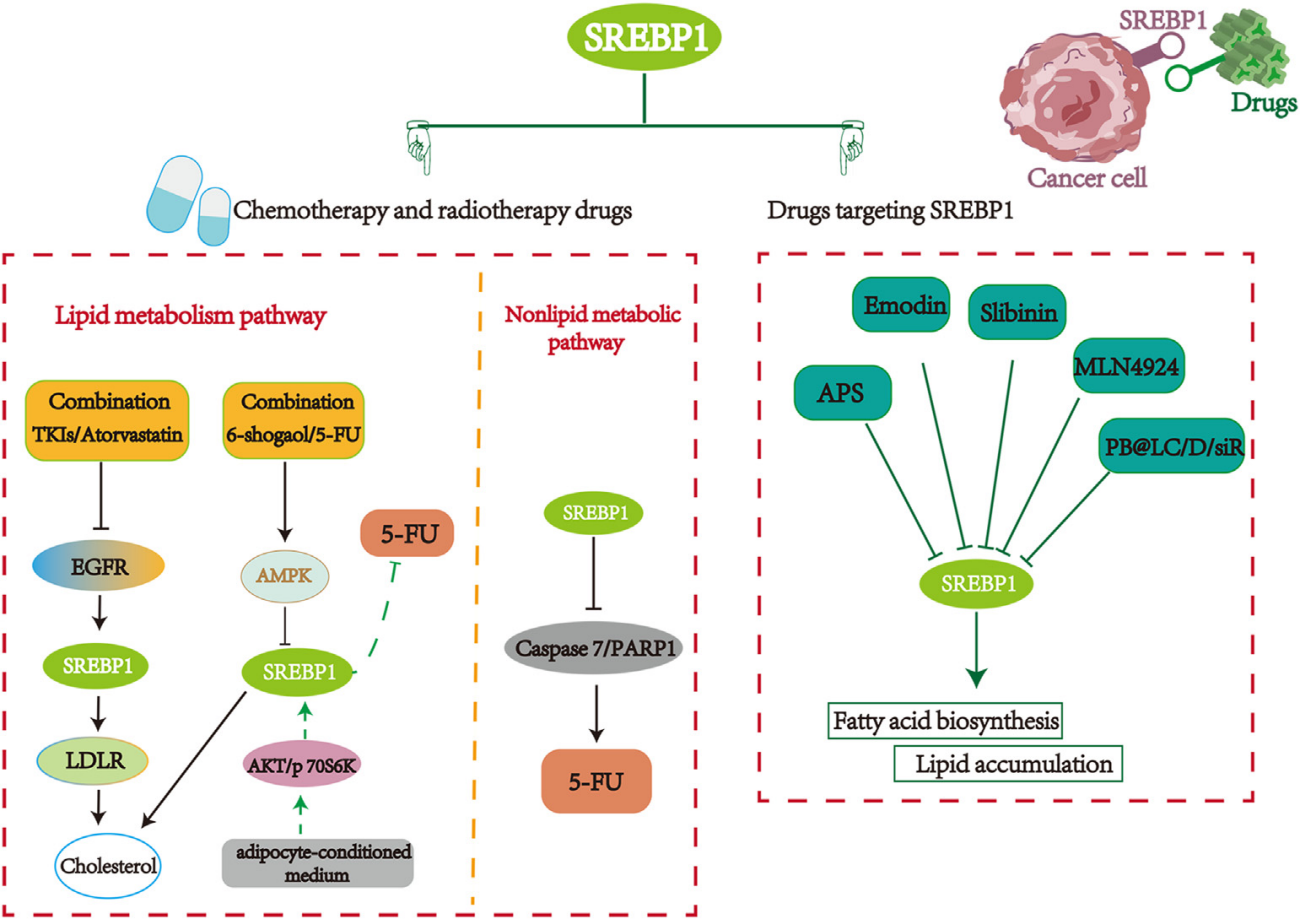
The regulation of SREBP1 in drug response. On the one hand, SREBP1 affects the sensitivity of cancer cells to chemotherapy drugs and radiotherapy by lipid metabolism pathway or non-lipid metabolism pathway. On the other hand, an increasing number of drugs targeting SREBP1 and the signaling pathway molecules have been explored.

During cancer treatment, acquired or innate resistance to chemotherapy and radiotherapy drugs is frequently observed, which remains a formidable obstacle.^64^^,^^65^ Accumulated evidence demonstrates that targeting SREBP1 and its associated signaling pathways can affect the sensitivity of cancer cells to chemotherapy and radiotherapy. For instance, blocking the SREBP1/FASN pathway in colorectal cancer could inhibit cholesterol synthesis and promote radiation-induced cell death.^66^ Meanwhile, low-density lipoprotein receptor (LDLR) exhibits a positive correlation with EGFR, which could up-regulate LDLR protein in an SREBP1-reliant manner. Tyrosine kinase inhibitor (TKI) decreases LDLR levels by inhibiting the EGFR/SREBP-1 pathway. Compared to the single drug treatment, the combination of EGFR-TKI and atorvastatin have a greater tumor suppressive effect in non-small cell lung cancer, suggesting that the combination medicine (statin and TKI) has the potential to be a novel therapy for EGFR-mutant non-small cell lung cancer (Fig. 4).^67^ Furthermore, numerous mechanisms shed light on the emergence of 5-FU resistance in colorectal cancer.^68^ For instance, when colorectal cancer cells are cultured in the adipocyte-conditioned medium, raising SREBP1 expression through p70S6K, Akt signaling pathways decreases the sensitivity of colorectal cancer cells to 5-FU. Furthermore, 6-shogaol, a ginger derivative, could attenuate the effect of adipocyte-conditioned medium on colorectal cancer cells by activating AMPK signaling, indicating the potential for clinical application of 6-shogaol in colorectal cancer treatment (Fig. 4).^69^ Remarkably, SREBP1, as an important lipid metabolism factor, could also modulate the sensitivity of chemotherapeutic drugs through non-lipid metabolism manner. In colorectal cancer, SREBP1 promotes resistance of 5-FU through inhibition of caspase7 expression and reduction of PARP1 cleavage fragments, potentially providing a novel target in cancer therapy (Fig. 4).^70^ In addition, FBXw7 could increase the mutant IDH1 expression by preventing the SREBP1 degradation, while silencing FBXW7 results in IDH1-mutated tumor cells being highly responsive to radiotherapy.^71^

### Drugs targeting SREBP1 and the signaling pathway molecules

SREBP1 considerably contributes to tumorigenesis and cancer development and can regulate the sensitivity of chemotherapy and radiotherapy drugs. Data show that SREBP1 and its signal pathway proteins are vital targets in drug design and study (Table 2).

**Table 2:** Related drugs targeting SREBP1 and its signal pathway molecules.

Cancer types	Drugs	Mechanism	Refs
Prostate Cancer	APS	Inhibition of SIRT1 and SREBP1 expression levels after miR-138-5p knockdown	35
	Docetaxel (DTX)	Regulating abnormal lipid metabolism to enhance tumor inhibition and anti-metastatic abilities	77
	Valproic acid（VPA）	Inhibits cell viability via decreasing lipogenesis and inducing apoptosis via the C/EBPα/SREBP1 pathway	57
	EJCE	decreasing expression of SREBP1 and FASN，which was reduced the intracellular fatty acid levels and lipid droplet accumulation.	91
Hepatocellular carcinoma	Emodin	SREBP1, ACLY, ACACA and FASN are inhibited and fatty acid synthesis is reduced	73
	Betulin	Blocking SREBP1s transcription factor activity specifically, thereby suppressing the glucose metabolism of cells	40
Endometrial carcinoma	Silibinin	Inhibits STAT3 signaling activation and reduces the expression level of SREBP1 extremely downstream genes	75
Non-small cell lung cancer	Atorvastatin	Blocking the SREBP1-dependent EGFR signaling pathway, reducing the utilization of cholesterol and lead to tumor growth inhibition	67
	G-Rh2+ cyclophosphamide (CY)	Suppressing the expression and nuclear translocation of SREBP1, and disturbing the SREBP1–FASN interaction，which regulating fatty acid metabolism	92
Breast cancer	MLN4924	Inhibiting of SREBP1 neddylation	4
Clear cell renal cell carcinoma	betulin	Suppressing SREBP1-dependent lipid metabolism and cell cycle progression	86
Multiple myeloma	MG-132	The ATF4 was induced to bind to the promoter region of SREBP1, resulting in abnormal lipid accumulation	93
Pancreatic cancer	Fatostatin, PF429242	Inhibit the activation of SREBP1 and affect its downstream signals such as FAS, HMGCoAR, SCD-1 and p53	94
Colon cancer	Berberine	Inhibiting SREBP1 activation and SCAP expression，which was led to the suppressed lipid synthesis	95
Colorectal Cancer	6-Shogaol	Activating the AMPK signaling to attenuate the ACM effect on SREBP1 expression and 5-FU-induced cell death in DLD-1 CRC cells	69
	Ilexgenin A	Regulating lipid metabolism through SREBP1 by the suppression of HIF-1α.	66
	TVB - 2640	Blocking SREBP1/FASN axis will enhance the curative effect of radiotherapy for colorectal cancer patients.	66

Astragalus polysaccharide is the primary active component of Astragalus, which has been linked to diverse biological processes, including metabolism, inflammation, and carcinogenesis.^72^ It suppresses the invasion and proliferation of prostate cancer cells and decreases cellular triglyceride and cholesterol levels. It may also negatively regulate SIRT1, inhibiting SREBP1 expression and nuclear translocation by activating AMPK phosphorylation to inhibit lipid metabolism (Fig. 4).^35^ The major active component of He Shou Wu (in Pinyin), emodin, could induce apoptosis and reduce the desaturation and triglyceride levels of FAs in HCC. Moreover, emodin is an effective inhibitor of SREBP1 and its target signaling pathway proteins, including FASN, ACLY, and ACACA, ultimately leading to reduced FA biosynthesis (Fig. 4).^73^ Likewise, silibinin is a flavonoid (flavonoid polyphenol) of natural origin isolated from the fruit of the chrysanthemum plant *Silybum marianum*, having antioxidant and antitumor properties. Silibinin contributes to various biological processes, including cell proliferation, apoptosis, and angiogenesis, and exhibits promising potential cancer treatment.^74^ Silibinin decreases STAT3 expression and its downstream genes involved in the cell cycle and apoptosis, reduces SREBP1 expression in the nucleus, and lessens the lipid accumulation in the endometrial carcinoma cells (Fig. 4).^75^ Furthermore, MLN4924, a definite inhibitor of the NEDD8-activating enzyme-E1, exerts significant anti-cancer effects primarily through the initiation of cellar apoptosis, senescence, and autophagy (Fig. 4).^76^ Heo et al demonstrated that MLN4924 down-regulates SREBP1 levels and its downstream genes such as FAS, ACC, and SCD-1.^4^ In addition, similar with paclitaxel, docetaxel is first-line chemotherapy for a patient with bone metastatic castration-resistant prostate cancer. Chen et al synthesized a nanodelivery system (PB@LC/D/siR) for the highly targeted release of docetaxel and siSREBP1 on the bone metastatic niche of such prostate cancer. By regulating abnormal lipid metabolism, the combination of siSREBP1 and docetaxel showed increased tumor inhibition and anti-metastasis capabilities (Table 2).^77^ Numerous additional SREBP1-targeting drugs are listed in Table 2.

### Challenges and opportunities

Metabolic abnormalities are important risk factors in many diseases, such as nonalcoholic steatohepatitis, liver fibrosis, fatty liver, and obesity.^78^, ^79^, ^80^ The protein expression of SREBP1 could be regulated by several post-translational modifications such as neddylation, phosphorylation, ubiquitination, and SDMA, regulating SREBP1 stability in cancer cells. Some of these modifications share a similar modification process but have distinct biological functions. Notably, neddylation modification is similar to ubiquitination, but unlike ubiquitination; neddylation labels NEDD8 onto the substrate protein, which does not degrade directly but modulates the activity/function of the protein.^81^ The UBC12 mediate the neddylation of SREBP1. Knockdown of UBC12 or its inhibitor MLN4924 could impair the tumor lipid metabolism pathway, confirming that UBC12-mediated SREBP1-like ubiquitination promotes tumor progression. In addition, neddylation inhibits ubiquitination modifications, but their mechanism remains unexplored.^4^ Therefore, to understand the effects of different post-translational modifications of SREBP1 protein in tumor cells and the relationship between each other, it is necessary to assemble and collate the latest advances related to SREBP1 modifications and investigate other post-translational modifications of proteins (glycosylation, nitrosylation, acetylation, and lipidation) for their mechanisms in modification and regulation of SREBP1.

Metabolic reprogramming, a hallmark of malignancy, plays a vital part during tumor initiation and progression.^82^ Increasing evidence shows that SREBP1 acts as a crucial oncogenic factor during tumorigenesis and cancer progression by regulating signaling pathways involved in lipid metabolism. Since FAs are limited in the human body for fast-growing tumors, many tumor cells synthesize unsaturated FAs by *de novo* synthesis pathway, modulated by several key enzymes such as ACLY, SCD, FASN, and ACC. The up-regulation of SREBP1 and these lipid synthases is commonly observed in cancer tissues and cells. Therefore, in-depth research into the mechanism of SREBP1 regulating lipid metabolism is a new direction for investigating novel clinical therapies. Meanwhile, lipid metabolism displays a significant correlation with glucose and mitochondrial metabolism. Since cells with a high level of glucose and energy can transfer into lipids, high glucose augments triglyceride synthesis and SREBP1 expression. Mitochondria are the primary site of ATP generation and oxidative phosphorylation in cells, and SREBP1 knockdown leads to a reduction in glycolysis, mitochondrial metabolism, and FA oxidation.^83^

SREBP1 plays an oncogenic role in immunity and TME, EMT, cell cycle, and programmed cell death and renders tumor cells therapeutically resistant. Currently, enzymes and transcription factors involved in lipid metabolic pathways are potential cancer drug targets. SREBP1 is a key intracellular transcription factor regulating lipid metabolism, and therefore numerous drugs targeting SREBP1 and the related signaling pathways can facilitate treating the tumors. Evidence indicates that targeting SREBP1 can improve cancer cells' sensitivity to various anti-cancer agents, and combination therapy, such as SREBP1-related inhibitors and chemotherapeutic agents, can enhance the sensitivity to chemotherapy and radiotherapy drugs to inhibit tumor proliferation and metastasis. Advances in high-throughput screening technologies have greatly contributed to the further understanding of SREBP1, and SREBP1-targeting compounds are anticipated to be effective combination drugs. When utilizing SREBP1-targeted compounds in future research, several obstacles must be considered. Screening for more targeted and effective inhibitors should be enhanced, as the number of selective or specific inhibitors of the SREBP1 regulator is still limited.

In conclusion, SREBP1 is a master transcription gene that modulates lipid reprogramming by regulating *de novo* lipogenesis and lipid homeostasis in cancer cells. Thus, a further comprehensive study of its expression regulation, roles in various cell processes, signal pathways, and interacting key regulators involved, or its effect on the sensitivity of chemotherapy and radiotherapy still necessitates further research to understand SREPB1 and its potential clinical application comprehensively.

## Author contributions

Ying He, Shasha Qi, Lu Chen, and Shuiping Liu drew the figures and wrote and revised the manuscript. Jinyu Zhu, Linda Liang, Xudong Chen, Hao Zhang, Lvjia Zhuo, and Shujuan Zhao reviewed and edited the manuscript. Shuiping Liu and Tian Xie contributed to the concept and design of the study and revision of the manuscript. All the authors read and approved the final manuscript.

## Conflict of interests

The authors declare no conflict of interests.

## Funding

This work was supported by the 10.13039/501100001809National Natural Science Foundation of China (No. 81802371), 10.13039/501100004543China Scholarship Council (No. 201908330151), 10.13039/501100004731Zhejiang Provincial Natural Science Foundation (China) (No. LQ17H160009), 10.13039/501100017594Zhejiang Province Medical Science and Technology Project (China) (No. 2018KY108, 2021RC117), Zhejiang Traditional Chinese Medicine Scientific Research Fund Project (China) (No. 2022ZB230), Hangzhou Health Science and Technology Major Project (Zhejiang, China) (No. Z20230119), and Hangzhou Agricultural and Social Development Scientiﬁc Research Independent Application Project (Zhejiang, China) (No. 20191203B22).
